# New Hypothesis and Alternative Approach for Imaging Neuronal Function and Metabolic Activity Based on Redox-Status

**DOI:** 10.4274/balkanmedj.2018.0286

**Published:** 2018-05-29

**Authors:** Plamen Getsov, Zhivko Zhelev, Ichio Aoki, Rumiana Bakalova

**Affiliations:** 1 Department of Radiology, Sofia University “St. Kliment Ohridski” School of Medicine, Sofia, Bulgaria; 2 Department of Radiology, “Tsaritsa Yoanna-ISUL” University Hospital, Sofia, Bulgaria; 3 Trakia University School of Medicine, Stara Zagora, Bulgaria; 4 Institute of Biophysics and Biomedical Engineering, Bulgarian Academy of Sciences, Sofia, Bulgaria; 5 Department of Molecular Imaging and Theranostics National Institute of Radiological Sciences, QST, Chiba, Japan; 6 Group of Quantum-state Controlled MRI, National Institute of Radiological Sciences, Chiba, Japan

To the Editor,

This article is intended to bring about a change in the way of thinking about the molecular nature of functional magnetic resonance imaging (fMRI) (1). The underlying assumption in our hypothesis is that fMRI may report mitochondrial activity in the synaptic area of the activated neuron, as well as an increased nitric oxide synthase activity at the same locus. The hypothesis is based on the uniqueness of the structure and functioning of the activated synapse and the major biochemical consequences of this structural–functional phenomenon.

The synapse is the primary energy source in the neuron, and the secret of fMRI might be hidden in the processes occurring in this locus.

***What is unique in the synapse?*** It contains a large amount of neurotransmitter vesicles, as well as a large amount of mitochondria due to the huge energy demand in this part of the neuron. In the synaptic mitochondria, 34-38 molecules of adenosine triphosphate (ATP) per molecule of glucose are synthesized based on glucose metabolism in the tricarboxylic acid (TCA) cycle (Figure 1) (2,3). It must be emphasized that “activity-driven ATP synthesis” in the synapse occurs for a short time in a very limited space – only in the area of the activated neuron.

***What are the consequences of the “activity-driven ATP synthesis” in the synaptic mitochondria?*** Electrons are derived through the mitochondrial electron transport chain (ETC), and a proton gradient is established across the inner mitochondrial membrane as an energy source for ATP synthesis. The most important biochemical event, associated with this process, is the production of a large amount of superoxide in the activated synapse for a very short time, due to the large amount of metabolically active mitochondria in this locus (Figure 2a). It must be clarified that this is not a permanent hyperproduction of superoxide, which can cause oxidative stress in the neuron. This is just a momentary event, which is precisely controlled by various factors (ETC complexes, antioxidant enzymes, uncoupling proteins, etc.) (3,4).

Superoxide radicals are paramagnetic species that are detectable directly by imaging techniques such as MRI (5). Moreover, hyperproduction of superoxide in metabolically active synaptic mitochondria may affect either T_1_ and/or T_2_ relaxation times of the surrounding protons. This will change the ^1^H MRI contrast in the activated synapse for a very short time – a well-known principle of enhancement of MRI contrast by other radicals (e.g., nitroxides) (6).

Тo clarify the role of superoxide in the relaxivity of water molecules, we performed a very simple MRI experiment in a pure chemical system, deionized water (DIW) plus potassium superoxide. Potassium superoxide rapidly decomposes into superoxide radicals in water, which is accompanied by enhancement of T_1_ and T_2_ MRI contrast (Figure 2b).

Superoxide is also an intermediate involved in the paramagnetic or diamagnetic transformations of hemoglobin during the transport of oxygen in the activated neuron (7). The hemoglobin transformations are considered as the primary factors for BOLD signals in fMRI measurements.

It is interesting to note that nitric oxide (NO), derived from NO adducts (as nitroglycerine or NOC9) in DIW, also increased T_1 _and T_2_ MRI contrast. However, the enhancement disappeared very rapidly, and it was difficult to obtain reproducible data due to the time required for MRI setting (data are not shown). NO is one of the major mediators of neurovascular coupling, and its local concentration in the activated synaptic area must also be high (8). The hyperproduction of NO and superoxide is spatially separated – production of superoxide occurs in the synaptic mitochondria, whereas that of NO occurs in the vascular endothelium. This eliminates the possibility of interaction between NO and superoxide with the formation of toxic peroxynitrite.

*The described data suggest that local and relatively long-lasting hyperproduction of superoxide by “activity-driven mitochondria” and NO in the synaptic area may be a major factor, changing proton relaxivity, and a root cause of the fMRI signals.* Therefore, fMRI can be used not only as a tool for detecting neural activity but also for detecting other metabolically active cells and tissues (e.g., cancer).

We believe that the hypothesis could be proved theoretically and experimentally using Overhauser-enhanced MRI (OMRI) based on proton–electron cross-relaxation phenomenon (9). However, the OMRI hybrid instruments are still “home-made,” expensive, and not available on the market. In addition, the experimental verification of the hypothesis requires a unique team of specialists – neurophysiologists, biochemists, biophysicists, specialists in molecular imaging, and mathematicians. This article aims to provoke the creation of such teams and to set their efforts in a direction that can open new trends in the concept of “functional imaging.” The described hypothesis could have a significant impact in all areas of molecular and functional imaging using MRI, positron emission tomography, optical, etc.

## Figures and Tables

**Figure 1:**
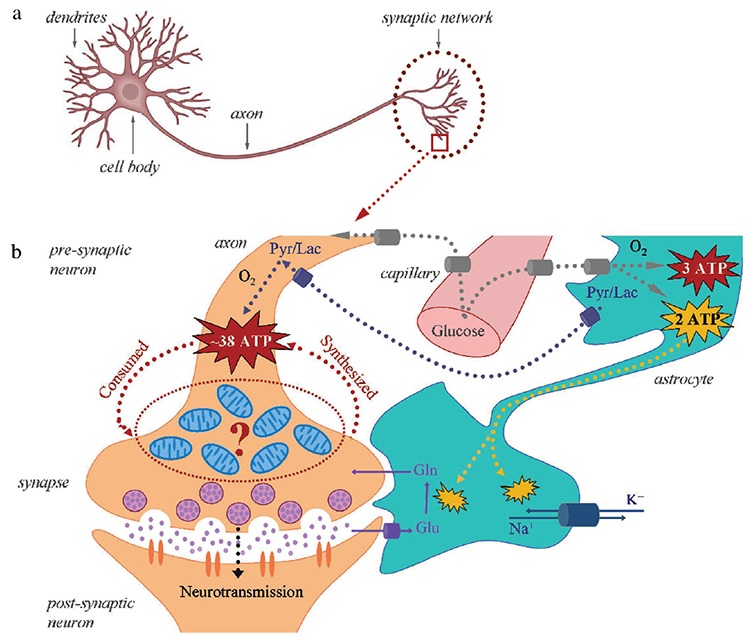
General structure of neuron with an emphasis on the synaptic area (a), General structure of synaptic area illustrating the coupling between synaptic activity, glucose metabolism, and energy supply during neural stimulation. The emphasis is on the presence of a large amount of mitochondria in the synapse and the high consumption of adenosine triphosphate and its synthesis de novo. Adenosine triphosphate (ATP) (in red) is produced in the tricarboxylic acid cycle, while ATP (in yellow) is produced in the glycolysis (b). *Gln: glutamine; Glu: glutamate; Lac: lactate; Pyr: pyruvate [Adapted according to Hyder et al. (2)]*

**Figure 2:**
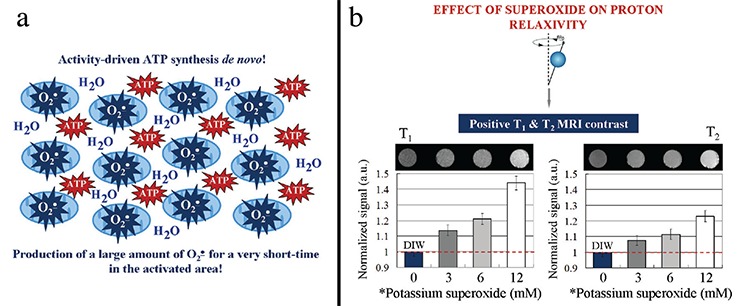
“Activity-driven adenosine triphosphate (ATP) synthesis” in the synapses and production of a large amount of superoxide radicals for a very short time in the activated synaptic area – in close proximity to water molecules (a), Effect of superoxide on T_1_ and T_2_ relaxation times of deionized water (DIW) (gradient echo magnetic resonance images). Magnetic resonance images (MRI) and corresponding graphs obtained from phantoms with different concentrations of potassium superoxide are presented (b). Experimental conditions: Time between dissolution of potassium superoxide in DIW and start of the MRI measurement was ~10 min. T _2_ images were obtained at TR= 3000 ms, TE= 90 ms, FA= 180°, FOV= 5.12, MTX= 256, and total scan time= 12 min 48 s. T_1_ images were obtained at TR= 20 ms, TE= 9.6 ms, FA= 180°, FOV= 5.12, MTX= 256, NA= 10, and total scan time= 10 min 40 s. *The concentrations of potassium superoxide on the x-axis must correspond to very low concentrations of superoxide (below 1 μM) at the start of MRI measurement, based on the assumption that potassium superoxide decomposes instantly and completely into superoxide radical, which is converted into hydrogen peroxide with a rate constant of ~10^5^ M^-1^.s^-1^. Representative images of phantoms are shown in the figure. In the graphs, the data indicate mean ± standard deviation from six independent experiments. The data were calculated by *ParaVision* software.
